# A Novel Magnetic Resonance Imaging Score Predicts Neurodevelopmental Outcome After Perinatal Asphyxia and Therapeutic Hypothermia

**DOI:** 10.1016/j.jpeds.2017.09.043

**Published:** 2018-01

**Authors:** Lauren C. Weeke, Floris Groenendaal, Kalyani Mudigonda, Mats Blennow, Maarten H. Lequin, Linda C. Meiners, Ingrid C. van Haastert, Manon J. Benders, Boubou Hallberg, Linda S. de Vries

**Affiliations:** 1 Department of Neonatology, Wilhelmina Children's Hospital, University Medical Center Utrecht, Utrecht University, The Netherlands; 2 Department of Neonatology, Karolinska Institutet and Karolinska University Hospital, Stockholm, Sweden; 3 Department of Radiology, Wilhelmina Children's Hospital, University Medical Centerer Utrecht, Utrecht University, the Netherlands; 4 Department of Radiology, University Medical Centre Groningen, Groningen, The Netherlands

**Keywords:** MRI, score, hypoxic-ischemic encephalopathy, hypothermia, outcome, ^1^H-MRS, Proton magnetic resonance spectroscopy, ADC, Apparent diffusion coefficient, BSITD-III, Bayley Scales of Infant and Toddler Development, third edition, CP, Cerebral palsy, DWI, Diffusion-weighted image, GMFCS, Gross Motor Function Classification System, HIE, Hypoxic-ischemic encephalopathy, MRI, Magnetic resonance imaging, NAA, *N*-acetyl aspartate, WISC-IV-SV, Wechsler Intelligence Scale for Children, fourth edition, Swedish version, WPPSI-III-NL, Wechsler Preschool and Primary Scale of Intelligence, third edition, Dutch version

## Abstract

**Objective:**

To assess the predictive value of a novel magnetic resonance imaging (MRI) score, which includes diffusion-weighted imaging as well as assessment of the deep grey matter, white matter, and cerebellum, for neurodevelopmental outcome at 2 years and school age among term infants with hypoxic-ischemic encephalopathy treated with therapeutic hypothermia.

**Study design:**

This retrospective cohort study (cohort 1, The Netherlands 2008-2014; cohort 2, Sweden 2007-2012) including infants born at >36 weeks of gestational age treated with therapeutic hypothermia who had an MRI in the first weeks of life. The MRI score consisted of 3 subscores: deep grey matter, white matter/cortex, and cerebellum. Primary adverse outcome was defined as death, cerebral palsy, Bayley Scales of Infant and Toddler Development, third edition, motor or cognitive composite scores at 2 years of <85, or IQ at school age of <85.

**Results:**

In cohort 1 (n = 97) and cohort 2 (n = 76) the grey matter subscore was an independent predictor of adverse outcome at 2 years (cohort 1, OR, 1.6; 95% CI, 1.3-1.9; cohort 2, OR, 1.4; 95% CI, 1.2-1.6), and school age (cohort 1, OR, 1.3; 95% CI, 1.2-1.5; cohort 2, OR, 1.3; 95% CI, 1.1-1.6). The white matter and cerebellum subscore did not add to the predictive value. The positive predictive value, negative predictive value, and area under the curve for the grey matter subscore were all >0.83 in both cohorts, whereas the specificity was >0.91 with variable sensitivity.

**Conclusion:**

A novel MRI score, which includes diffusion-weighted imaging and assesses all brain areas of importance in infants with therapeutic hypothermia after perinatal asphyxia, has predictive value for outcome at 2 years of age and at school age, for which the grey matter subscore can be used independently.

Although therapeutic hypothermia after perinatal asphyxia has reduced the incidence of adverse outcome, 45% of infants still die or have neurodevelopmental impairment.^1^, ^2^, ^3^, ^4^, ^5^, ^6^, ^7^ Magnetic resonance imaging (MRI) and proton magnetic resonance spectroscopy (^1^H-MRS) have been shown to be excellent predictors of outcome^4^, ^8^, ^9^, ^10^, ^11^ and are often used as bridging biomarkers for neurodevelopmental outcome in infants with hypoxic-ischemic encephalopathy (HIE).^12^, ^13^ Quantifying the extent of brain injury in these infants is important for objective and accurate prognostication and guiding decisions on redirection of care. Many existing MRI scores do not include diffusion-weighted images (DWI),^4^, ^9^, ^10^, ^14^ even though DWI has been shown to be the most reliable MRI sequence to assess injury during the first week after an hypoxic-ischemic event.^15^ Early detection is important for the selection of future additional neuroprotective strategies, which may need to be initiated within the first week after birth. Some abnormalities encountered on MRIs of infants with HIE, such as intracranial hemorrhages, cerebellar lesions, and MRS abnormalities are not part of existing scores, although they may be of additional value. We developed a novel score based on assessment of all MRI abnormalities of suspected importance for prognostication in infants with HIE. We hypothesized that our novel MRI score, which includes DWI as well as assessment of the deep grey matter, white matter, and cerebellum, will have a better predictive value for neurodevelopmental outcome at 2 years of age and at school age than conventional MRI-based scoring systems that have been described previously.

## Methods

The ethics committees of both participating centers waived the requirement to obtain informed consent for this retrospective study with anonymized data. Infants born after a gestational age of >36 weeks admitted to a level III neonatal intensive care unit in the Netherlands (cohort 1, January 2008-March 2014; n = 97) and Sweden (cohort 2, January 2007–December 2009; n = 76) treated with therapeutic hypothermia for HIE (defined as a Thompson score of >7 and/or a discontinuous electroencephalograph) owing to presumed perinatal asphyxia (5-minute Apgar score ≤5, pH ≤7.10, base deficit ≥16 mmol/L, or resuscitation 10 minutes after birth) and examined by brain MRI as part of routine clinical care were included. Infants with major congenital abnormalities, inborn errors of metabolism, and genetic syndromes were excluded. In both centers whole-body cooling was started as soon as possible within 6 hours after birth and continued for 72 hours. After 72 hours, the babies were rewarmed gradually to 36.5°C. After rewarming, the babies were kept at a temperature of 36.5°C for 12-24 hours.

In both cohorts, MRI was performed on a 1.5-T or 3.0-T magnet (Philips Medical Systems, Best, The Netherlands, GE Healthcare, Chicago, Illinois), within the first weeks after birth. Standard MRI protocol included axial T1-weighted images or inversion recovery-weighted images, T2-weighted images, DWI including apparent diffusion coefficient (ADC) mapping. Only in cohort 1 were ^1^H-MRS measurements in the basal ganglia and thalamus performed (details published previously).^8^

**Figure 1:**
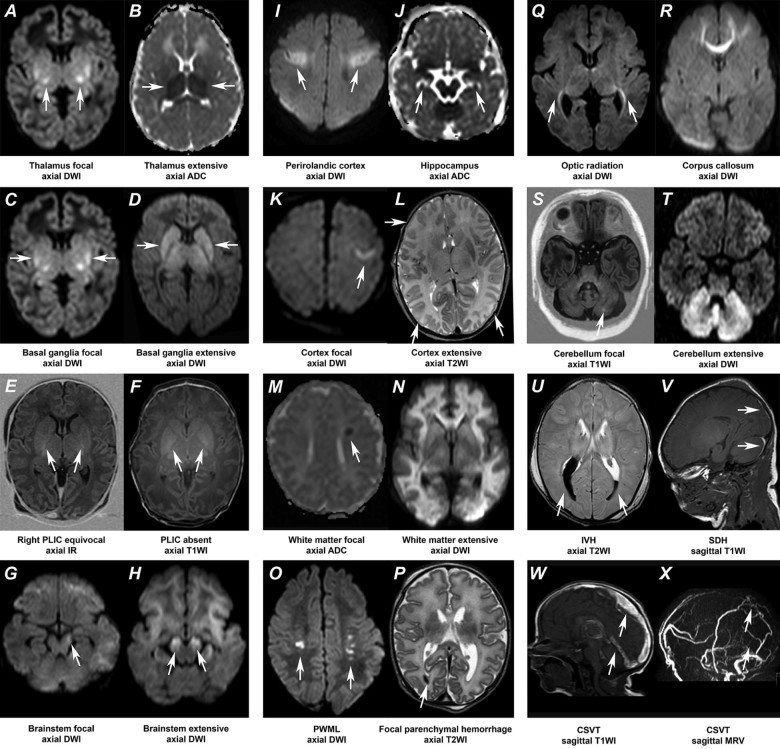
MRI examples of all items to be scored with the novel MRI score. The abnormalities of interest are marked by the *white arrows*. **A,** Focal bilateral thalamic lesions (high signal intensity [SI]) on an axial DWI. **B,** Extensive bilateral thalamic lesions (low SI) on an axial ADC map. **C,** Focal bilateral lesions (high SI) in the basal ganglia on an axial DWI. **D,** Extensive bilateral lesions (high SI) in the basal ganglia on an axial DWI. **E,** The posterior limb of the internal capsule (PLIC) is equivocal on both sides on an axial inversion recovery (IR) image. **F,** Absent PLIC bilaterally seen as an inverted signal (low SI) on an axial T1-weighted image (T1WI). **G,** Focal lesion (high SI) in the left cerebral peduncle on an axial DWI. **H,** Extensive diffusion changes (high SI) in the cerebral peduncles bilaterally on an axial DWI. **I,** Clear involvement (high SI) of the perirolandic gyrus bilaterally on an axial DWI. **J,** Bilateral involvement (low SI) of the hippocampus on an axial ADC map. **K,** Focal involvement (high SI) of the left cortex on an axial DWI. **L,** Extensive bilateral involvement of the cortex, seen as loss of the differentiation between the white matter and cortical grey matter in the occipital and frontal lobes bilaterally. **M,** Focal unilateral abnormal signal (low SI) in the left periventricular white matter on an axial ADC map. **N,** Extensive involvement of the white matter (high SI) on an axial DWI. **O,** Bilateral punctate white matter lesions (PWML) seen as high SI on an axial DWI. **P,** A small focal hemorrhage in the right occipital lobe (low SI) on an axial T2-weighted image (T2WI). **Q,** Bilateral involvement of the optic radiation (high SI) on an axial DWI. **R,** Involvement of the frontal part of the corpus callosum (high SI) on an axial DWI. **S,** Focal lesion (high SI) in the left cerebellar hemisphere on an axial T1WI. **T,** Extensive involvement of both cerebellar hemispheres (high SI) on an axial DWI. **U,** Bilateral intraventricular hemorrhage (IVH) seen as low SI on an axial T2WI. **V,** Subdural hemorrhage (SDH) supra- and infratentorial seen as high SI on a sagittal T1WI. **W,** Cerebral sinovenous thrombosis (CSVT) seen as high SI at the location of the superior sagittal and straight sinus on a sagittal T1WI. **X,** With corresponding lack flow (lack of high SI) in those veins on an MR venography (MRV) in sagittal view.

The MRI score was designed based on previously published scores and the patterns of brain injury reported in the literature (Table I; available at www.jpeds.com*)*.^10^, ^13^, ^14^ Our score assesses brain injury in 3 areas: (1) deep grey matter (items scored: thalamus, basal ganglia, posterior limb of the internal capsule, brainstem, perirolandic cortex, and hippocampus; maximum grey matter subscore, 23), (2) cerebral white matter/cortex (items scored: cortex, cerebral white matter, optic radiations, corpus callosum, punctate white matter lesions, and parenchymal hemorrhage; maximum white matter subscore, 21), and (3) cerebellum (items scored: cerebellum and cerebellar hemorrhage; maximum cerebellum subscore, 8). Each item was scored for extent of injury: 0 (no injury), 1 (focal, <50%), or 2 (extensive, ≥50%) and for unilateral (score of 1) or bilateral (score of 2) presence, the items were not weighted at this stage. A fourth group (additional) was included, assessing the presence of intraventricular or subdural hemorrhages and sinovenous thrombosis 0 if absent, 1 if present. The maximum additional subscore was 3. The total score was calculated by adding the 4 subscores (grey matter + white matter + cerebellum + additional; maximum score, 55). In cases where ^1^H-MRS was performed in the basal ganglia and thalamus, *N*-acetyl aspartate (NAA) and lactate were scored 0 (normal NAA peak, absent lactate peak) or 1 (reduced NAA peak, increased lactate peak), which was subsequently included in the grey matter subscore (maximum grey matter subscore, 24; total score, 57). The ADC measurements were performed when visual analysis of the ADC map was inconclusive, and items were scored as abnormal if the ADC values in the specific area were lower than previously defined cutoff values.^8^ MRI examples for each item of the score are shown in Figure 1.

Two reviewers blinded to patient outcomes assessed all MRIs using the score described above. In case of disagreement consensus was obtained with a third blinded reviewer. To determine inter-rater reliability, 2 additional blinded pediatric radiologists (one local to Utrecht one external to the institutions scored the injury on MRI on a subset of scans (n = 10).

### Neurodevelopmental Outcome

The Bayley Scales of Infant and Toddler Development, third edition (BSITD-III), was used to assess outcome at 2 years.^16^ The Wechsler Preschool and Primary Scale of Intelligence, third edition, Dutch version (WPPSI-III-NL)^17^ and Wechsler Intelligence Scale for Children, fourth edition, Swedish version (WISC-IV-SE)^18^ was used to assess IQ at school age (cohort 1, 5.5-6.5 years; cohort 2, 6.5-8 years). Severity of cerebral palsy (CP) was classified according to the Gross Motor Function Classification System (GMFCS).^19^ For infants with CP who could not be tested with the BSITD-III, a motor composite score was assigned, 70 (-2 SD on the BSITD-III) for GMFCS III, and 45 (-3 SD) for GMFCS IV-V. For infants with severe CP (GMFCS V) who could not be tested with the BSITD-III, a cognitive composite score and for the WPPSI-III-NL or WISC-IV-SE a total IQ score of 45 was assigned. Abnormal outcome was defined at 2 years as death, CP (GMFCS ≥ II), or a BSITD score <85 (-1 SD) for motor or cognitive composite score, and at school age as death, CP (GMFCS ≥ II) or an IQ <85.

### Statistical Analyses

GraphPad Prism 6 (GraphPad Software Inc, La Jolla, California) was used to generate receiver operating characteristic curves, calculate the area under the curve, and determine the cutoff values for the MRI score (total and subscores) based on the point on the receiver operating characteristic curve closest to the (0,1) point.

Cronbach alpha was used to determine the intraclass correlation coefficient, as a measure of inter-rater reliability for the total score and all subscores. Sensitivity, specificity, positive predictive value, negative predictive value, and accuracy (total number of correctly predicted individuals [true positive + true negative/all observations × 100]) were calculated. SPSS 21 (IBM Corp, Armonk, New York) was used to determine differences between the 2 cohorts in baseline characteristics using the Mann Whitney U test and the χ^2^ or Fisher exact test and to perform univariate and multivariable logistic regression to investigate the association between adverse outcome, the BSITD-III and IQ scores, and the MRI subscores. BSITD-III and IQ scores were dichotomized for logistic regression, with 85 (−1 SD) being the cutoff level for adverse outcome. *P* < .05 was considered significant.

## Results

MRI scans from cohort 1 were used to develop the score and scans from cohort 2 to validate it. In cohort 1, there were 27 infants with a normal scan and in cohort 2, there were 16 infants. The majority of scans were obtained in the first week after birth (81%). The quality of the scans was good (no movement artefacts, high resolution) in 85.6% for cohort 1 and 71.4% for cohort 2. The baseline characteristics are shown in Table II. Cohort 2 had a higher gestational age at birth, higher birth weights, lower Apgar scores at 5 minutes, and fewer deaths, but a higher survival rate with impairment at 2 years of age compared with cohort 1. One infant with CP had a GMFCS II (cohort 1), and the other infants with CP had a GMFCS ≥ III (all cohort 2); the GMFCS level did not change between 2 years of age and school age.

**Table II:** Baseline characteristics of the 2 cohorts

	Cohort 1 n = 97	Cohort 2 n = 76	*P* value
Gestational age (wk), mean (SD)	39.9 (1.6)	40.3 (1.4)	.060
Birth weight (g), mean (SD)	3497 (610)	3708 (662)	.039
Male, n (%)	53 (54.6)	36 (47.4)	0.342
Apgar at 5 min, median (IQR)	4 (2-5)	3 (2-4)	.032
Sarnat grade, n (%)*			.056
1	12 (12.4)	4 (5.3)	
2	67 (69.1)	62 (81.6)	
3	18 (18.6)	7 (9.2)	
MRI			
MRI age (day of life), median (IQR)	6 (5-7)	6 (5-8)	0.315
Total score, median (IQR)	6 (0-22)	3 (1-11.8)	0.122
Grey matter subscore	0 (0-11.5)	0 (0-4.5)	
White matter subscore	4 (0-8)	2 (0-7)	
Cerebellum subscore	0 (0-4)	0 (0-0)	
Outcome			
Died, n (%)	22 (22.7)	5 (6.6)	.004
Age at BSITD-III assessment in months, mean (SD)	24.13 (0.42)	25.92 (1.68)	<.001
BSITD-III motor composite score, mean (SD)	112 (12)	95 (23)†	<.001
BSITD-III cognitive composite score, mean (SD)	107 (14)	95 (21)	<.001
Age at IQ assessment in years, mean (SD)	5.9 (0.3)	7.5 (0.8)	<.001
IQ, mean (SD)	102 (17)‡	100 (19)§	0.555
Impairment at 2 years, n (%)	4 (4.1)	14 (18.4)	.029
BSITD-III motor composite score < 85	2 (2.1)	13 (17.1)	
BSITD-III cognitive composite score < 85	2 (2.1)	10 (13.2)	0.773
Impairment at school age, n (%)	4 (7.5)†	5 (10.9)§	
CP	1 (1.9)	4 (8.7)	
IQ < 85	3 (5.7)	4 (8.7)	

* Data available for 73 subjects in cohort 2.

† Data available for 57 subjects.

‡ Data available for 53 subjects.

§ Data available for 46 subjects.

Cronbach alpha was 0.95 for the total score without ^1^H-MRS (0.96 including ^1^H-MRS), 0.98 for the grey matter subscore with and without ^1^H-MRS, 0.94 for the white matter subscore, 0.72 for the cerebellum subscore, and 0.89 for the additional subscore.

### MRI Score and Neurodevelopmental Outcome

At 2 years of age, outcome data were available for all infants in both cohorts. The grey matter, white matter, and cerebellum subscores were significantly associated with death or adverse outcome in both cohorts and were subsequently included in a multivariable analysis. The multivariable regression model included grey matter subscore as an independent predictor of death or impairment at 2 years of age in cohort 1 (model without ^1^H-MRS: OR, 1.6, 95% CI, 1.3-1.9 [ß_0_ = −4.579, ß_1_ = 0.456]; model with ^1^H-MRS: OR, 1.6, 95% CI, 1.3-1.9 [ß_0_ = −5.017, ß_1_= 0.443]) and cohort 2 (model without ^1^H-MRS: OR 1.4, 95% CI 1.2-1.6 [ß_0_ = −2.310, ß_1_ = 0.322]).

At school age (cohort 1, mean of 5.9 years; cohort 2, mean of 7.5 years), outcome data were available for 53 infants in cohort 1: 22 died and 31 had follow-up assessment. For 44 infants, no follow-up information was available at school age: 35 were too young, 1 was not testable owing to behavioral problems, and 8 were not tested for unknown reasons. In cohort 2, outcome data at school age were available for 46 infants: 5 died and 41 had follow-up assessment. For 30 infants, no follow-up information at school age was available: 19 were too young and 11 were not tested for unknown reasons. There were no differences in baseline characteristics between infants with follow-up assessment and those lost to follow-up. The grey matter and white matter subscores were significantly associated with death or adverse outcome in both cohorts. The grey matter, white matter, and cerebellum subscores were included in a multivariable analysis. The multivariable regression model included the grey matter subscore as an independent predictor of death or impairment at school age in both cohort 1 (model without ^1^H-MRS: OR, 1.3, 95% CI, 1.2-1.5 [ß_0_ = −2.394, ß_1_ = 0.292], model with ^1^H-MRS: OR, 1.3, 95% CI, 1.1-1.5 [ß_0_ = −2.333, ß_1_ = 0.262]) and cohort 2 (model without ^1^H-MRS: OR, 1.3, 95% CI, 1.1-1.6 [ß_0_ = −2.747, ß_1_ = 0.286]).

The grey matter subscore was significantly correlated with the white matter and cerebellum subscore in both cohorts (*P* < .001). Entering white matter and/or cerebellum subscores in the models resulted in a reduction in the OR of the grey matter subscore, suggesting multicollinearity.

**Figure 2:**
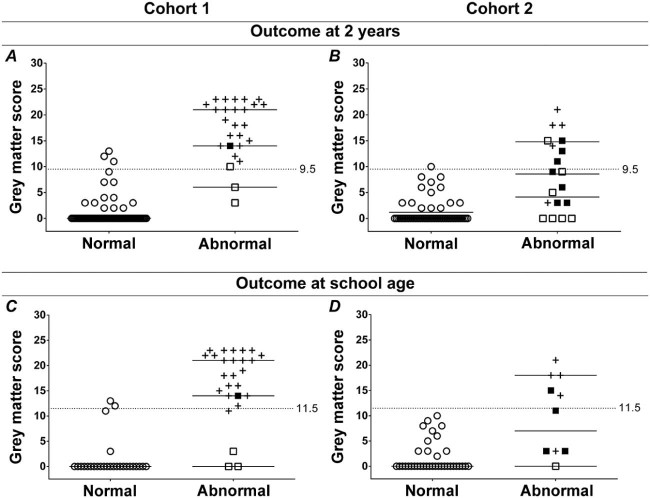
Individual score values on the grey matter subscore for infants with a normal (open circles) and infants with an abnormal outcome (death, black crosses; CP, black squares; other impairment, open squares) **A, B,** at 2 years of age; and **C, D,** at school age; **A, C,** in cohort 1; and **B, D,** cohort 2. The black horizontal lines indicate the median. The dotted horizontal lines indicate the cutoff values for risk of adverse outcome.

**Figure 3:**
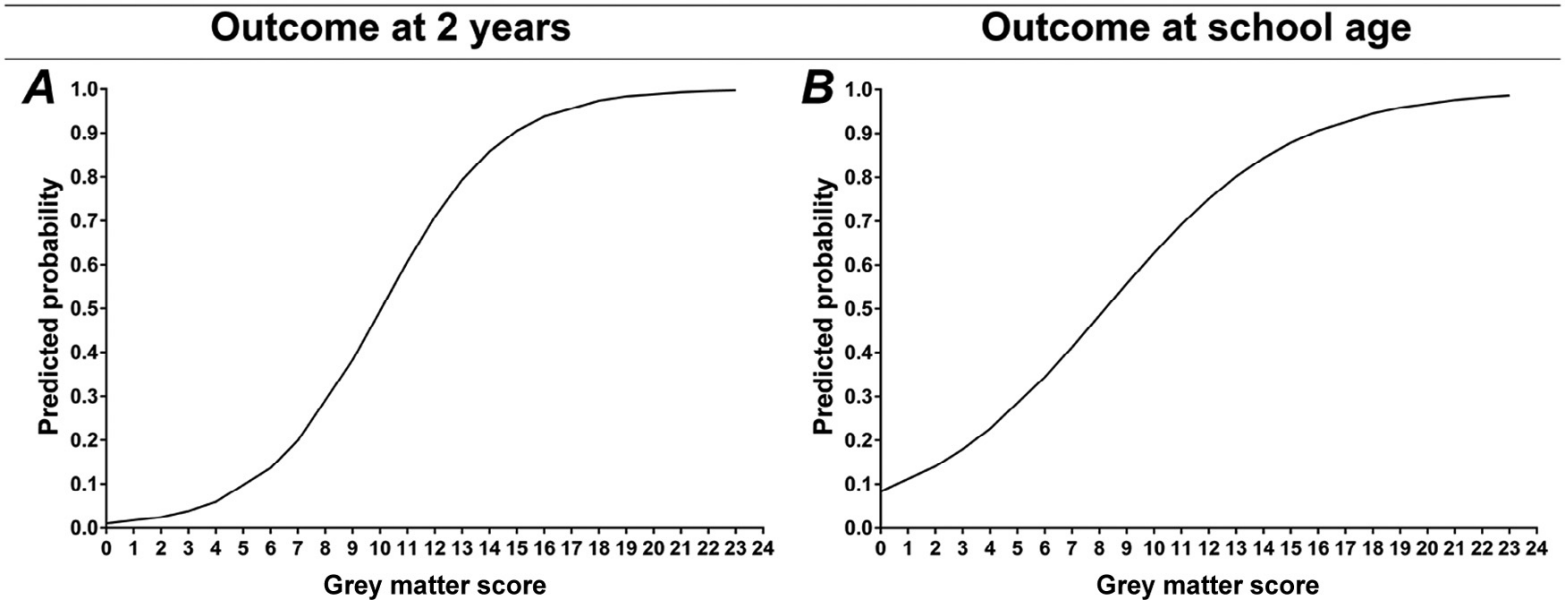
Predicted probability of death or impairment **A,** at 2 years of age and **B,** at school age based on the grey matter subscore in cohort 1.

Receiver operating characteristic curves for adverse outcome at 2 years of age and at school age were plotted for the grey matter subscore because injury to the grey matter subscore was an independent predictor of outcome. The area under the curve values with 95% CIs and sensitivity and specificity for the cutoff values are shown in Table III. Figure 2 shows the distribution of the individual scores in all infants with a normal vs an abnormal outcome at 2 years of age and at school age in both cohorts. A predicted probability graph for adverse outcome at 2 years of age and at school age was generated for the grey matter subscore in cohort 1 (Figure 3; available at www.jpeds.com).

**Table III:** Cross-tabulation of the MRI score results*

Score	Diagnostic accuracy								
	Cutoff	Normal	Abnormal	AUC (95% CI)	Sensitivity	Specificity	PPV	NPV	Accuracy
Cohort 1									
Outcome at 2 y									
Grey matter without ^1^H-MRS	<9.50	68	2	0.988 (0.973-1.000)	0.923	0.958	0.889	0.971	0.948
Grey matter without ^1^H-MRS	≥9.50	3	24						
Grey matter including ^1^H-MRS	<11.50	61	2	0.989 (0.973-1.000)	0.923	0.953	0.889	0.968	0.944
Grey matter including ^1^H-MRS	≥11.50	3	24						
Outcome at school age									
Grey matter without ^1^H-MRS	<11.50	25	4	0.945 (0.878-1.000)	0.846	0.926	0.917	0.862	0.887
Grey matter without ^1^H-MRS	≥11.50	2	22						
Grey matter including ^1^H-MRS	<12.50	20	3	0.935 (0.855-1.000)	0.885	0.909	0.920	0.870	0.896
Grey matter including ^1^H-MRS	≥12.50	2	23						
Cohort 2									
Outcome at 2 y									
Grey matter without ^1^H-MRS	<9.50	56	11	0.832 (0.708-0.955)	0.421	0.982	0.889	0.836	0.842
Grey matter without ^1^H-MRS	≥9.50	1	8						
Outcome at school age									
Grey matter without ^1^H-MRS	<11.50	36	5	0.861 (0.726-0.997)	0.500	1.000	1.000	0.878	0.891
Grey matter without ^1^H-MRS	≥11.50	0	5						

*AUC*, Area under the curve; *NPV*, negative predictive value; *PPV*, positive predictive value.

* Based on the optimal cutoff values for the grey matter subscore.

#### Adverse Outcome at 2 Years of Age and at School Age in Surviving Infants

The relationship between the MRI scores and the BSITD-III motor and cognitive composite scores at 2 years or the IQ at school age was assessed on the pooled cohort (cohorts 1 + 2), because the number of surviving infants with impairment was too small in the separate cohorts. At 2 years of age, the BSITD-III motor composite score was not available for 14 infants and a motor composite score was assigned for 5 infants with CP in cohort 2. At school age, an IQ score was assigned for 3 infants with CP in cohort 2. The grey matter and white matter subscores were significantly associated with a motor or cognitive composite score of <85 at 2 years of age. None of the subscores was significantly associated with an IQ <85 at school age. The multivariable logistic regression model included the grey matter subscore as an independent predictor of motor (model without ^1^H-MRS: OR, 1.3, 95% CI, 1.2-1.5 [ß_0_ = −3.126, ß_1_ = 0.294]) and cognitive impairment (model without ^1^H-MRS: OR, 1.3, 95% CI, 1.2-1.5 [ß_0_ = −3.504, ß_1_ = 0.290]) at 2 years of age. No analyses were performed on the scores, including ^1^H-MRS results, because these were only available for cohort 1.

## Discussion

We developed an easily applicable, comprehensive MRI score that showed good predictive value in 2 independent, international cohorts, comprising a total of 173 infants treated with therapeutic hypothermia. In both cohorts, injury to the deep grey matter area was an independent predictor of adverse outcome at 2 years of age and at school age. The grey matter subscore may be useful for outcome prediction in hypothermia-treated infants with HIE. The presented cutoff values and predicted probability graphs could be used to aid clinical decision-making or as an outcome measure in clinical trials. The inter-rater reliability was high and the predictive value remained good in cohort 2.

Most previously published MRI scoring systems that have been related to outcome^9^, ^10^, ^14^ were designed to be performed using T1- and T2-weighted images only and therefore use scans obtained in the second week of life, because abnormalities on these sequences may not yet be present in the first week.^20^ The predictive properties of our score are comparable with these previously published scores.^9^, ^10^, ^14^ Our score has the advantage of including DWI and can, therefore, be used in the first week of life, a period when important clinical decisions may have to be made and during which additional neuroprotective therapies could be initiated. Another advantage of our scoring system is that we use a point-by-point form with clear descriptions of what is considered moderate or severe injury, which can even be used by less experienced MRI readers. The scoring systems published by Barkovich et al, Rutherford et al (TOBY trial [Total Body Hypothermia for Neonatal Encephalopathy]), and Shankaran et al (National Institute of Child Health and Human Development [NICHD]) do not use an item-based scoring system, but group patterns of injury together.^9^, ^10^, ^14^ In our experience, it is sometimes difficult to score infants who have injury who do not fit the categories exactly. Additionally, the NICHD scoring system puts basal ganglia/thalamic injury and white matter injury in the same severity grade, although they do not have the same implications for outcome.^21^, ^22^, ^23^, ^24^ Four previously published scoring systems included DWI and target scans obtained in the first week of life as well.^4^, ^25^, ^26^, ^27^ The score presented by Jyoti et al had good predictive value, but included only 20 infants with a follow-up time of only 12 months.^25^ Conclusions about the predictive value of that score should therefore be considered with caution. Furthermore, the Jyoti score, similar to the NICHD score, also puts basal ganglia/thalamic injury and white matter injury in the same severity grade. The score presented by Cheong et al had a good specificity, but its sensitivity was suboptimal (0.68).^28^ Cavalleri et al used the summation score presented previously by Barkovich et al on DWI.^14^^,^^26^ This score showed a high sensitivity (1.00) but a lower specificity (0.67) and was based on ADC measurements, which are more complex and time consuming. The recently published score by Trivedi et al, which was weighted for grey matter injury, had a lower area under the curve of 0.72 (95% CI, 0.57-0.86), sensitivity of 0.77, and specificity of 0.46.^27^

Our results from a population of infants treated with therapeutic hypothermia confirm that injury to the deep grey matter is associated with adverse outcome in general and impaired motor function. White matter injury was not included in the prediction model for outcome at 2 years of age, because many infants with a high white matter subscore also had a high grey matter subscore. Only the grey matter score was included, suggesting that outcome was determined mainly by injury to the grey matter. These results support the findings of Harteman et al.^23^ We found no association between the white matter subscore and IQ at school age, which is different from other reports in the literature.^21^, ^29^, ^30^, ^31^ However, these studies were performed in normothermic infants and included infants with isolated severe white matter injury. In our cohort, infants with isolated white matter injury had only mild to moderate lesions, which did not have a significant impact on their cognition. For other populations, such as normothermic infants or infants with metabolic or infectious disorders, the score can be used to perform a complete assessment of the brain and quantify injury. However, the predictive value of the score will be different and needs to be ascertained for each population separately. Cerebellar injury was also not included in the prediction model, even though it was related to poor outcomes in cohort 1. Again, many infants with a high cerebellum subscore also had a high grey matter subscore, suggesting that deep grey matter injury is more important for outcome prediction than cerebellar injury. Besides, the majority of cerebellar injury was a rather unspecific increased signal on T2-weighted imaging, only 5.7% had a cerebellar hemorrhage.

We were, unfortunately, unable to assess and compare the quality of the score in the first vs the second week of life, because only a limited number of MRIs were performed in the second week of life. During the first week of life, it is best to perform the score based on the T1- and T2-weighted images combined with DWI, because DWI has been shown to be the most reliable sequence to assess injury in HIE in the first week of life.^15^ In the second week of life DWI abnormalities may no longer be visible owing to pseudonormalization.^32^ The optimal time window for performing DWI in our opinion would be between days 4 and 7. At this point, rewarming has been completed and DWI abnormalities will have reached their full extent,^33^ and pseudonormalization will not yet have occurred. However, the score can also be performed in the second week using T1- and T2-weighted images only. ADC and ^1^H-MRS measurements (lactate and NAA) in the basal ganglia can add significantly to the predictive properties of MRI. In contrast with ADC, which shows pseudonormalization after the first week,^32^ ^1^H-MRS measurements remain abnormal for a prolonged period of time.^34^

The limitations of our study are the lack of ^1^H-MRS measurements in cohort 2; thus, the score including ^1^H-MRS measurements still requires validation in another cohort. Furthermore, not all infants had follow-up at school age, which could have led to sampling bias. A significant difference in age at WPPSI/WISC assessment was seen between cohorts 1 and 2; however, regression analysis (data not shown) showed no relation between age at assessment and IQ. A difference in mortality and survival with impairment was observed between cohorts 1 and 2 as well. However, the proportion of infants with adverse outcome, either death or impairment, was exactly the same. In cohort 1, more infants died owing to redirection of care, but this was compensated by a higher number of infants that survived with impairment in cohort 2. The OR for prediction of adverse outcome was, therefore, not affected and remained stable in both cohorts and when infants who died were excluded. The MRI magnet strength was variable in this study, but we do not expect this factor to have influenced our results because magnetic field strength has been demonstrated not to affect ADC or ^1^H-MRS values.^8^ Most of our MRIs were performed in the first week after birth, and the predictive value of the score in the second week after birth still needs to be assessed. The sensitivity of the grey matter subscore was not as good in cohort 2 compared with cohort 1 (0.42 at 2 years of age and 0.50 at school age), yet the specificity, positive predictive value, negative predictive value, and accuracy remained good (>0.84). The reduction in sensitivity might be explained by the greater proportion of MRIs of moderate to poor quality in the test cohort. A poor quality MRI could result in an underestimation of the brain lesions and a lower sensitivity, underlining the importance of a good quality MRI. Although white matter and cerebellum injury did not have additional predictive value in our cohort, these factors could well be predictive in preterm infants, and other high-risk newborns. Scoring systems often perform quite differently in other populations. We should, therefore, always be careful when using scoring systems in other populations.

Ideally, the predictive value of scores and the reliability and applicability of cutoff values should be determined for each population separately. It is also possible that with a larger number of subjects or another cohort with a different distribution of injury, there may be a subset of HIE infants with predominant watershed/white matter patterns (without significant basal ganglia/thalamic injury) that would relate to cognitive outcomes. Our scoring system has some theoretical advantages over other systems, but these have not been validated from our study because we could not test other scores on our cohort, because these scores were different in that they do not use DWI.

In summary, we developed a novel MRI score that includes DWI, assesses all relevant brain areas, and was tested in 2 independent, international cohorts. In infants with therapeutic hypothermia after perinatal asphyxia, the grey matter subscore with the presented cutoff values and predicted probability graphs may be of use in prognosticating outcome. We also provide additional evidence that in this population outcome is mainly determined by injury to the deep grey matter area, independent of lesions to other areas of the brain such as the white matter.

## Figures and Tables

**Table I:** MRI scoring form

	Items	Sequence used to assess injury	Degree		
Grey matter			0	1	2
1	Thalamus abnormal SI or diffusion restriction	T1/T2 DWI	No	Focal (<50%)	Extensive (≥50%)
	Specify location			Unilateral	Bilateral
2	Basal ganglia abnormal SI or diffusion restriction	T1/T2 DWI	No	Focal (<50%)	Extensive (≥50%)
	Specify location			Unilateral	Bilateral
3	PLIC myelination or diffusion restriction	T1/T2 DWI	Normal or no diffusion restriction	Equivocal/partially myelinated or partial (<50%) diffusion restriction	Absent myelination or extensive (≥50%) diffusion restriction
	Specify location			Unilateral	Bilateral
4	Brainstem (peduncles) abnormal SI or diffusion restriction	T1/T2 DWI	No	Focal (<50%)	Extensive (≥50%)
	Specify location			Unilateral	Bilateral
5	Perirolandic cortex diffusion restriction	DWI	No	Mild	Clear
	Specify location			Unilateral	Bilateral
6	Hippocampus diffusion restriction	DWI	No	Yes	
	Specify location			Unilateral	Bilateral
Grey matter subscore					
	Basal ganglia NAA	^1^H-MRS	Normal	Reduced	
	Basal ganglia lactate	^1^H-MRS	Absent	Increased	
Grey matter subscore (including ^1^H-MRS)					
	White matter/cortex		0	1	2
1	Cortex abnormal SI or diffusion restriction not being perirolandic cortex	T1/T2 DWI	No	Focal (1 lobe)	Extensive (>1 lobe)
	Specify location			Unilateral	Bilateral
2	White matter increased SI or diffusion restriction not being PWML	T1/T2 DWI	No	Focal (1 lobe)	Extensive (>1 lobe)
	Specify location			Unilateral	Bilateral
3	PWML	T1/T2, DWI, SWI	No	<6	≥6
	Specify location			Unilateral	Bilateral
4	Hemorrhage not being PWML	T1/T2, SWI	No	Single hemorrhage <1.5 cm	≥1.5 cm or multiple hemorrhages
	Specify location			Unilateral	Bilateral
5	Optic radiation diffusion restriction	DWI	No	Mild	Clear
	Specify location			Unilateral	Bilateral
6	Corpus callosum diffusion restriction	DWI	No	Yes	
White matter subscore					
	Cerebellum		0	1	2
1	Cerebellum abnormal SI or diffusion restriction	T1/T2 DWI	No	Focal (<0.5 cm)	Extensive (≥0.5 cm or multiple lesions)
	Specify location			Unilateral	Bilateral
2	Cerebellar hemorrhage	T1/T2, SWI	No	Single hemorrhage <0.5 cm	≥0.5 cm or multiple hemorrhages
	Specify location			Unilateral	Bilateral
Cerebellum subscore					
	Additional		0	1	2
1	IVH	T1/T2, SWI	No	Yes	
2	SDH	T1/T2	No	Yes	
3	CSVT	T1/T2, MRV	No	Yes	
Additional subscore					
Total score (grey matter + white matter + cerebellum + additional score)					

*CSVT*, Cerebral sinovenous thrombosis; *IVH*, intraventricular hemorrhage; *MRV*, magnetic resonance venography; *PLIC*, posterior limb of the internal capsule; *PWML*, punctate white matter lesions; *SDH*, subdural hemorrhage; *SI*, signal intensity; *SWI*, susceptibility weighted imaging.
